# The Effects of Physiotherapy Programmes, Aided by Virtual Reality Solutions, on Balance in Older Women: A Randomised Controlled Trial

**DOI:** 10.3390/jcm13216462

**Published:** 2024-10-28

**Authors:** Marek Zak, Tomasz Sikorski, Agata Michalska, Paulina Sztandera, Beata Szczepanowska-Wolowiec, Waldemar Brola, Daniel Courteix, Frederic Dutheil

**Affiliations:** 1 Institute of Health Sciences, Collegium Medicum, Jan Kochanowski University, Zeromskiego 5, 25-369 Kielce, Poland; tomasz.sikorski@ujk.edu.pl (T.S.); michalska.agata@ujk.edu.pl (A.M.); paulina.sztandera@ujk.edu.pl (P.S.); beata.szczepanowska-wolowiec@ujk.edu.pl (B.S.-W.); waldemar.brola@ujk.edu.pl (W.B.); 2 Laboratory of the Metabolic Adaptations to Exercise Under Physiological and Pathological Conditions (AME2P), Université Clermont Auvergne, 63000 Clermont-Ferrand, France; daniel.courteix@uca.fr; 3 Occupational and Environmental Medicine, CHU, 63000 Clermont-Ferrand, France; fdutheil@chu-clermontferrand.fr; 4 Physiological and Psychosocial Stress, Université Clermont Auvergne, CNRS, LaPSCo, 63000 Clermont-Ferrand, France

**Keywords:** virtual reality, dual-task, physical functional performance, balance, aged, exercise therapy

## Abstract

**Background:** Modern technologies are being applied to maintain and improve the functional performance of older adults. Fully immersive virtual reality (VR) combined with a scope of dual-task (DT) activities may effectively complement conventional physiotherapy programmes for seniors. The study aimed to compare the effectiveness of a fully immersive virtual reality (VR) environment combined with a scope of dual-task activities regarding balance in older women. **Methods:** Eighty women were recruited to the study protocol and, following randomisation, allocated to two equally sized groups, one pursuing conventional OTAGO exercises, the other one the VR-solutions-aided exercise programme combined with a scope of DT activities. Physiotherapy sessions spanned 6 weeks, each one lasting 60 min, three times a week, in both groups. **Results:** Homogeneity analysis of both study groups indicated no statistically significant differences at the first measurement point. After the intervention, both study groups achieved significantly improved scores on all tests. The VR + DT group obtained better results in dual-task gait and single-leg standing, whereas the greatest difference was observed during SLS CL (1.52 s vs. 2.33 s—difference 0.81 s 53.2% change, *p* = 0.001). The OTAGO group performed better in the TUG single-task gait (11.35 s vs. 12.60 s, *p* < 0.001) and in the Berg balance scale. **Conclusions:** The VR + DT training is effective in improving individual balance as well as in reducing fall risks. VR-assisted physiotherapy should complement conventional physiotherapy programmes (e.g., OTAGO). The benefits of applying VR solutions indicate that older women might well use this form of activity regularly under the guidance of a therapist or a family member.

## 1. Introduction

Enhancing individual functional performance is believed to be essential in both reducing the fall risk and improving balance in older adults. Regular exercise routines can significantly improve one’s physical performance. Furthermore, improved functional performance contributes to one’s greater independence as well as a sense of well-being in daily life [1].

The term balance refers to an individual sense of control and stability one feels over one’s body, affecting the ability to move, change body position, and perform various tasks [2]. Balance disorder can limit an older adult’s ability to perform activities of daily living (ADL) independently [3]. Many systems and bodily organs work together to maintain balance, including vision, the nervous system, muscles, and the balance centre in the brain and in the inner ear. Balance problems correlate strongly with incidental fall among seniors, especially those aged 75 and older, when they actually become most vulnerable to functional decline [4].

Regular physical activity, including strengthening, balance, body movement coordination, and postural stability exercises, whilst adopting different postures, can significantly affect the overall quality of life in seniors, offering them greater safety and relative independence from third-party assistance [5,6]. Universal therapeutic programmes of proven efficacy, e.g., OTAGO, have been used worldwide in the therapeutic management of older adults for many years [7].

In recent years, two modern methods, i.e., dual-task training and exercises aided by virtual reality (VR) solutions, have been supplemented into conventional programmes aimed specifically at enhancing individual functional performance [8,9]. Patients (including the seniors) respond positively to VR-aided exercises whilst admitting to high-level concentration throughout. Overall, they rate this VR-assisted regimen as a most enjoyable and totally novel experience that actually prompts them to keep on exercising [10].

Published research offers ample evidence for the overall effectiveness of VR technological solutions in the rehabilitation of older adults. Studies are dominated by the application of VR solutions in incomplete immersion, whereas a vast majority of them are based on non-VR head-mounted display (HMD) systems like Nintendo Wii or Microsoft Kinect [11]. One of the key premises of VR is that it is meant to evoke natural as well as real sensations in the participating individuals by creating a diversity of stimuli for the person immersed in the virtual world to interact with, just in the same way as they would in the real world. In the last decade, the area of neurological rehabilitation has borne witness to the greatest increase in both general interest and academic research in the application of VR technological solutions [12].

The present study aimed to compare the overall effectiveness of VR HMD solutions juxtaposed against the application of conventional rehabilitation regimens, with a view to demonstrating whether VR HMD training, combined with a scope of dual-task activities, can offer an effective therapeutic method in addressing functional performance, individual balance issues, and fall risk in older adults. 

## 2. Materials and Methods

### 2.1. Study Design 

Subjects were recruited into 2 intervention groups. Potential subjects were advised about the upcoming opportunity to participate in the rehabilitation programme through the Senior Activity Centres network. The study was designed as a randomised control trial with single-blinding of the sample, relying on the subjects not knowing which specific rehabilitation group they belonged to and what training was being provided in the other group. The sample size was calculated using the Online Sample Size Calculator. Women were randomly allocated to two equally sized groups (OTAGO and VR + DT) using Excel.

Approval for this research experiment was granted by the Bioethics Committee of Jan Kochanowski University in Kielce. Furthermore, the registration of the study was made with the Australian New Zealand Clinical Trials Registry (ID No ACTRN12621000719831). The study was conducted in full compliance with applicable terms of the Declaration of Helsinki, good clinical practices, applicable legislation in place, and the applied CONSORT 2010 Statement. Informed consent to participate in the study was collected from all subjects in writing prior to randomisation.

### 2.2. Participants

Out of the 92 subjects invited to participate in the study, 80 women who were not currently or historically taking any medication for depression met the inclusion criteria. The mean BMI was 26.09. Inclusion criteria were as follows: (1) age ≥ 75 years, (2) consent from an internist or geriatrician for each person to participate, (3) MMSE > 23, (4) Dizziness and Balance Screening Questionnaire score of 10 out of 12 possible, and (5) BBS > 38. Participants were not admitted into the study if they met any of the following exclusion criteria: (1) Eye diseases and dysfunctions preventing participation in the study, (2) Dizziness of neurological origin, (3) Functional limb shortening, (4) Dizziness and Balance Disorders Screening Questionnaire score below 10, (5) Parkinson’s disease, (6) Unstable cardiovascular disease, (7) History of stroke, (8) Lack of informed written consent to participate in the study protocol, (9) Use of orthopaedic equipment during walking, (10) Medical conditions preventing participation, (11) Concurrent participation in another improvement programme, and (12) No written consent to participate.

### 2.3. Procedures

**Figure 1:**
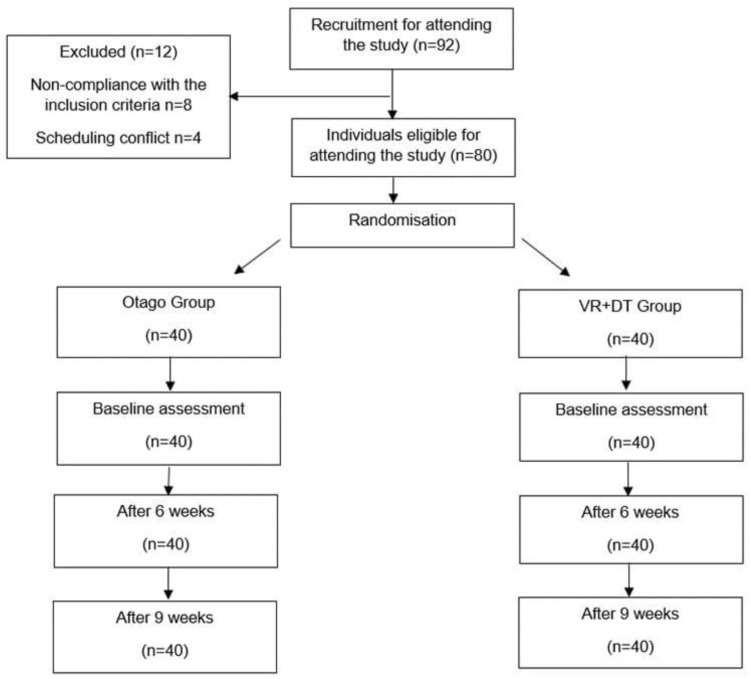
Recruitment process and the sizes of respective study groups.

The study protocol is presented in Figure 1.

### 2.4. Baseline Assessment

During recruitment to the study, a detailed interview was conducted with potential subjects regarding vital personal data, the general health status, current medical conditions, as well as any chronic diseases whose potential recurrence during the study might affect individual ability to participate in the study or perform in the fitness tests. At this stage, the possibility of dizziness, individual mental state, and balance were also examined, whereas the willing participants were asked to sign off a voluntary consent form. Our study envisaged no gender breakdown, with a view to examining the overall effects of the proposed intervention in older adults. Nevertheless, only women happened to be willing to attend the study protocol upon the actual recruitment, so the study protocol had to be modified accordingly.

Dizziness and Balance Disorders Screening Questionnaire—the questionnaire consists of 12 questions with the option to answer YES or NO to each, determining the presence of symptoms rather than their severity [13].

Mini-mental-State Examination (MMSE)—designed to assess individual mental state and cognitive function, the MMSE is a 30-item questionnaire that assesses the patient’s cognitive functions, including orientation, memory, attention, language, and visuospatial abilities [14].

Berg Balance Scale (BBS)—a balance assessment tool consisting of 14 movement tasks that are scored on a five-point scale (0–4). Tasks include standing on one leg and standing up from a chair and moving around, which effectively facilitates the assessment of both one’s static and dynamic balance [15].

The following tests were also applied at the first and subsequent measurement points to determine balance and fall risk:

Timed up and go test (TUG)—it involves assessing the balance and mobility of a patient who starts in a seated position in a chair and is asked to stand up, walk three metres, turn 180 degrees, and then return to his seat. The timing of the test and how the tasks are performed, including the need for arm support or stability when walking, are analysed to assess fall risk and balance [16].

Timed up and go test cognitive (TUG COG)—this modification of the TUG test was applied to assess the scope of dual-task motor-cognitive activities. The subject simultaneously performs an additional cognitive task during gait, such as counting backwards from a random number [17].

Timed up and go test manual (TUG MAN)—this modification of the TUG test was applied to assess the scope of dual-task motor-motor activities. The subject simultaneously performs an additional motor task during gait by carrying an object, such as a glass of water [17].

A Single-leg Stance Test (SLS)—both the open-eyed (SLS OP) and the closed-eyed (SLS CL) versions were applied, whereupon the subject was required to stand on one leg alone, with the hands resting on the thighs [18].

The following VR devices were used in the study protocol:

Carl Zeiss VR ONE plus-ZEISS^®^ goggles, which contain two aspherical biconvex lenses with +32.5 diopters, allow for the smartphone with the enabled application to be placed by sliding it into the front tray of the head-mounted system at a distance of 44 mm, allowing full 3D immersion for the subject.

OTAGO Group

The training plan for the OTAGO group was based on ‘The OTAGO Exercise Programme’ [19]. The training comprised both the sitting and standing positions, accompanied by the use of leg weight cuffs. After the warm-up, subjects performed a series of consecutive specific exercises (balance, flexibility and resistance training), which were rounded off with some stretching. The warm-up time was approximately 5 min, the specific training 50 min, and stretching 5 min. Training took place 3 times in each one of 6 consecutive weeks (total of 18 sessions, with 2 absences admissible).

VR + DT Group

A training plan for the group in dual-task and VR conditions was developed by the Author, based on the study by Liu et al., involving dual-task motor exercises (1) walking with putting a ball between the hands, (2) walking with tossing a ball, and cognitive exercises (3) talking while walking, (4) walking whilst adding up numbers, (5) walking whilst subtracting numbers, (6) walking whilst repeating phrases, (7) walking whilst reciting a word chain, and (8) walking whilst identifying objects. 

**Figure 2:**
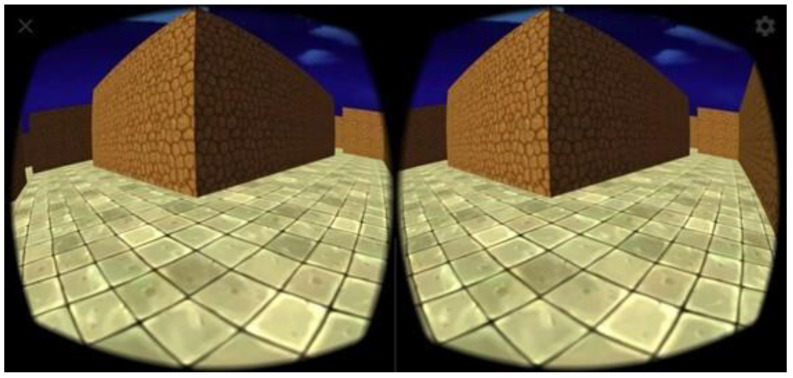
View of the game in VR goggles (from the subject’s perspective, merged into a single image).

In the first training session, questions required short answers, e.g., yes/no (Did you have breakfast today?) and in the following sessions, subjects were required to answer in the form of complex sentences (What did you have for breakfast today?). The difficulty level of the tasks in the following sessions was also graded, e.g., by increasing the number subtracted cyclically or by repeating phrases consisting of more words. Walking was performed forwards, backwards, and by following the shape of figure “eight”. The training plan was planned for six weeks with a frequency of three times a week. A single training session lasted approximately 60 min (5 min warm-up, 25–30 dual-task training, 25–30 virtual reality training). Training took place 3 times in each of 6 consecutive weeks. The VR app selected for this particular study is the freely available app., ‘VR Maze Walk Journey’ (Figure 2).

This application was deliberately selected with a view to creating a VR environment as part of the study protocol by Zak et al. [20]. The subject’s task was to traverse a randomly generated maze (with different levels of difficulty). The subject, immersed in the virtual world, was supposed to control his movements through appropriate head movements. Moving forward was also accomplished by indicating where to move with eye movement. The character stopped when the person looked down at an angle of at least 10 degrees from looking straight ahead (this was most often used by subjects to think about the actual route ahead and reflect on it). During the game, subjects had to perform rotations (also in a full 360 degrees) in order to pass the maze, and every 30 s, the researcher gave a command, on which the subject had to perform 2 half squats and a 5 s toe or heel raise. 

## 3. Results

Homogeneity analysis of the respective groups was carried out on the basis of variables such as age, gender, education, weight, height, BMI, number of falls in the past year, number of medications regularly taken, assessment of condition by MMSE, and balance level by BBS. No statistically significant differences between groups were encountered. Detailed characteristics are provided in Table 1.

Six weeks after the commencement of the study (i.e., the conclusion of the intervention), the VR + DT group scored better on the TUG MAN, TUG COG, SLS OP, and SLS CL tests compared to the OTAGO group. The OTAGO group scored significantly better on TUG (11.35 s vs. 12.60 s, *p* < 0.001) and BBS. All subjects enrolled in the study completed the study without reporting any adverse effects (Table 2).

Nine weeks after the commencement of the study (i.e., three weeks after the conclusion of the intervention), the OTAGO group scored better in TUG (12.26 vs. 12.82, *p* = 0.01), TUG MAN, TUG COG and BBS, as compared to the VR + DT group. The VR + DT group performed better in the SLS OP and SLS CL tests (Table 3).

A significant improvement was observed in all test scores in both groups. There were improvements in TUG between pre- and post-intervention results in the OTAGO group (13.45 s vs. 11.35 s—difference 2.1 s 15.6% change, *p* < 0.001) and the VR + DT group (14.02 s vs. 12.60 s—difference 1.42 s 10.1% change, *p* = 0.001), in TUG MAN for the OTAGO group (13.72 s vs. 12.42 s—difference 1.3 s 9.4% change, *p* < 0.001) and the VR + DT group (13.23 s vs. 11.62 s—difference 1.61 s 12.1% change, *p* = 0.001), in TUG COG for the OTAGO group (16.16 s vs. 14.60 s—difference 1.56 s 9.6% change, *p* < 0.001) and the VR + DT group (17.02 s vs. 14.13 s—difference 2.89 s 17% change, *p* = 0.001), in the BBS for the OTAGO group (39.33 pts. vs. 42.58 pts.—difference 3.25 pts. 8.2% change, *p* < 0.001) and the VR + DT group (39.68 pts. vs. 41.88 pts.—difference 2.2 pts. 5.5% change, *p* = 0.001), in SLS OP for the OTAGO group (5.39 s vs. 7.90 s—difference 2.51 s 46.5% change, *p* = 0.001) and the VR + DT group (8.81 s vs. 9.87 s—difference 1.06 s 12% change, *p* = 0.01), and in SLS CL for the OTAGO group (1.40 s vs. 2.07 s—difference 0.67 s 47.9% change, *p* < 0.001) and the VR + DT group (1.52 s vs. 2.33 s—difference 0.81 s 53.2% change, *p* = 0.001) (Table 4).

## 4. Discussion

The present study offers a pioneering approach to assessing the effects of a VR HMD + DT exercise regimen on overall functional performance in older adults. Our previous research [20] demonstrated that the application of just a few sessions utilising VR solutions and combined VR solutions + DT activities may well translate into a tangible improvement of the key variables of individual functional performance. This time around, however, the study’s principal focus was narrowed down to assessing individual balance and fall risk.

This study evaluated the effects of 18 training sessions incorporating functional performance exercises using fully immersive VR solutions combined with a scope of dual-task activities in older women ≥ 75 years, in comparison with conventional physiotherapy, i.e., the OTAGO exercise programme. The age criterion for enrolment in the study was adopted specifically in view of a small number of published studies on the application of fully immersive VR covering this specific age group, and, to the best of our knowledge, this study happens to be the first one in which the VR training is innovatively combined with a scope of dual-task activities. 

After the intervention, both groups achieved satisfactory improvements in the key variables of functional performance. A within-group analysis indicated that the VR + DT group achieved statistically significantly better results in improving functional balance, dual-task gait, and a reduction in fall risk (*p* = 0.001).

The results indicated an improvement in static balance against the SLS tests. The VR + DT group achieved the greatest improvement in the SLS CL test (out of all tests) after the intervention (53.2% change). Individual dynamic balance and mobility were assessed by TUG. An improvement was noted in both groups, although the scores in the OTAGO group proved better in the intergroup analysis upon the conclusion of the study protocol. Having said that, when assessing dual-task gait, it was the VR + DT group that proved to have performed better in the TUG COG (17% vs. 9.6%) and in the TUG MAN (12.1% vs. 9.4%). Moreover, the ability to maintain balance in different situations was assessed through BBS. Improvements were noted in both groups, with the OTAGO group scoring higher (3.25 points vs. 2.2 points). 

This change may not be relevant in clinical terms, however, as Donoghue et al. [21] propose that an improvement of ≥5 points is required to be 95% confident that a real change has occurred if an older adult’s BBS score has initially remained within the 35–44 points range. 

Different results were reported by Chen et al. [22], whose study showed that the control group scored lower than the VR group. On the other hand, the control group pursued a different form of training regimen, i.e., Tai Chi. In another study, Yoo et al. [23] reported a significant rise in the Berg balance scale scores (47.60 ± 5.36 before and 53.50 ± 2.30) following the subjects’ exposure to VR environment.

These data indicate that IVR training may well complement conventional exercise regimens specifically aimed at improving balance and reducing overall fall risk. When coupled with the dual-task exercises, which combine motor and cognitive tasks, it may be used interchangeably with the conventional programmes whilst offering a beneficial effect on the dual-task gait, as compared to the OTAGO programme in women aged 75 years and older.

Within 3 weeks of the intervention’s conclusion, it was noted that the participants’ performance continued to improve compared to prior to completing the programme. This indicates that the intervention has a long-lasting effect on improving individual functional performance. A study by Rebêlo et al. [24] indicates that the effects of IVR training persist even after 2 months, covering various aspects of individual balance, as indicated by the DGI test. Nevertheless, much as in the present study, the results in both groups were inferior to those achieved immediately after the intervention. This may indicate that the initial benefits of the intervention tend to diminish over time.

Even though our programme required the assistance of a physiotherapist while performing the tasks within the IVR setting (owing to the cut-off of visual stimuli), it is crucial to maintain the long-term effects of the training and transform those benefits into a lasting improvement. This may well be achieved through continuing the pursuit of the programme in one’s own home environment, with some assistance from immediate family members. It is also feasible to have one’s posture modified during the pursuit of IVR exercises into a sitting position on a chair, which allows for rotation whilst reducing overall fall risk.

Despite the OTAGO group having scored higher in the TUG, the VR + DT group achieved greater improvement in the dual-task modifications of this test. This is most likely due to the very nature of the intervention applied. The VR group exercised under dual-task conditions, i.e., both by way of performing an additional manual task during gait and a cognitive one by having to face a specific mental task. 

Furthermore, the application of immersive VR techniques for balance training represents an innovative approach that is well grounded in biomechanical as well as psychological foundations. The key component consists of overall postural control, which is essential for maintaining individual balance. This training engages multimodal feedback, which includes a variety of senses, e.g., vision, hearing, touch, as well as vestibular and proprioceptive senses. This enables participants to gain a better understanding of their own body and its position in space, which is crucial for maintaining effective balance control.

Adequate awareness and perception of one’s body plays a key role in motor learning. Participants need to be aware of their body movements and their impact on balance, which is supported by the VR techniques providing instant feedback. The application of VR technology solutions makes it possible to simulate different scenarios, which in turn increases individual attention span and reaction time and facilitates the performance of dual tasks, which is essential in individual balance training. An individual sense of immersion in a virtual environment is crucial for effective learning. The participants, feeling part of the virtual world, are more likely to engage in the exercises and much keener on experimenting within a new environment. 

Peterson et al. [25] indicate that likely visual perturbations naturally occurring within the IVR setting induce cortical responses in the occipital and parietal regions and may improve the brain’s ability to adapt to the changes taking place in the sensory stimuli. Such immersion promotes better acquisition of motor skills, which is an essential factor in the rehabilitation and training of older adults remaining at risk of neurological and cardiovascular incidents and/or immobilisation due to injury or hospitalisation [26].

In the study by Sadeghi et al. [27] on the effects of differently designed types of training regimens aimed at improving lower limb muscle strength, balance, and functional mobility in older men, both a VR group and a mixed exercise group attested to significant therapeutic benefits. The VR group subjects scored better in balance and functional mobility, as compared to a conventional balance training group and a control group, respectively. On the other hand, the mixed exercise group, combining conventional balance training with the VR-aided exercise regimen, reported the greatest improvements in lower limb muscle strength, balance, and functional mobility, thus highlighting the occurrence of a substantial synergistic effect between the differently designed exercise regimens. These results are closely similar to the ones secured in our own study, which combined the VR-aided solutions with a scope of dual-task exercises rather than with a conventional balance training regimen, even though the overall level of complexity of our own research project, well-grounded in highly customised, modular VR environment was significantly more advanced, pending re-development and further expansion, mostly in view of highly encouraging, therapeutic outcomes.

It should be duly noted at this juncture that VR-aided solutions are also being investigated with regard to their therapeutic potential in addressing different disorders. In a study by Ozkul et al. [28], the authors applied a VR-aided intervention in individuals affected by multiple sclerosis (MS) and compared the outcomes to the ones obtained in a study group pursuing a conventional regimen aimed at a control group for enhancing individual balance. The results attained by both study groups improved, being also closely similar statistically.

In a study by Jung et al. [29], on the other hand, the training was conducted with stroke survivors. The group training with VR-aided solutions scored significantly better than the control group in terms of TUG scores. VR-aided solutions may also be successfully applied in patient diagnostics, as they affect the timing of the TUG test and the actual number of steps made by the subjects exposed to a fully immersive VR environment [30].

The results of the present study demonstrated improvements in various variables, including a notable reduction in fall risk through a more effective and confident gait in the TUG test. Fear of falling does not only affect older adults who have actually experienced such incidents. After 75, the fear of falling over often leads to immobility and perceptively reduced functional performance, and consequently, diminished overall quality of life [31]. 

This fear may largely be attributable to the muscular and reactive demands of moving around in different environments, e.g., walkways, shopping areas, or apartments. In such situations, it is necessary to use continuous balance strategies that include anticipating potential threats and implementing various compensatory actions [32]. It is of particular importance to such individuals to be offered an array of structurally attractive exercises so they can be encouraged to fully engage in such specifically targeted physical activity whilst feeling safe at all times. 

Research by Dockx et al. [33] indicates that training in the VR setting is perceived as extra attractive to older adults, as opposed to conventional training methods. Participation in VR-assisted programmes offers seniors a chance to engage in an interactive and much-varied type of training experience, consequently keeping them highly motivated throughout. The high appeal of the VR setting also consists in the fact that it may easily be tailored to the individual needs and abilities of its potential users. Moreover, another added value is that older adults may pursue their exercises in a safe, controlled environment, which alleviates their overall concern about sustaining an incidental fall.

No significant medical complications or adverse effects were reported in the study participants during exposure to the virtual environment, whereas the IVR + DT programme proved to be an effective and safe method for functional performance training. The application of IVR settings to improve individual balance whilst reducing fall risk in older adults is also supported by other studies [20,24,34,35,36]. The combined IVR + DT programme also maximises the therapeutic effects, owing to the enhanced brain neuroplasticity, naturally stimulated by the need to adapt to the new challenges which require concurrent information processing [37].

Individual balance training, aimed at preventing incidental falls, requires a complex approach involving both muscle strengthening and sensory stimulation. Making use of a single method only is insufficient in terms of addressing effectively all the key aspects [38,39]. Our self-designed rehabilitation programme combines these components in direct response to a variety of diagnosed problems in older adults, including individuals over 75 years of age. It is well grounded in a complementary combination of dual-task and IVR exercises, translating not only into physical performance but also into the training of body coordination, balance, sensory stimulation, and improvement of large and small motor skills when executing precision movements required during the pursuit of IVR + DT exercise regimen.

Significant functional changes were noted following the completion of 18 group therapy sessions (60 min each) by the study participants, i.e., manifest improvement in individual functional performance, which is well corroborated by the outcomes of the previously published studies by other authors. But first and foremost, the combined VR + DT regimen perceptibly helps to normalise all body movement initiation/termination paradigms in older adults, which in turn holds positive implications for individual balance whilst reducing overall fall risk. 

This innovative, combined VR + DT regimen also helps the participants modify all ineffective body movements accordingly or even avoid them altogether when attending to their daily routines. 

Very frequently, older adults also tend to demonstrate a fairly limited ability to learn any new tasks and/or make use of the newly mastered body movement paradigms when performing their daily living activities. This cognitive deficit is quite effectively addressed by the application of this innovative VR + DT regimen, which also perceptibly improves individual balance. 

### Study Limitations

The present study is also subject to some limitations, nevertheless. Firstly, the study did not have a control group unassigned to any specific intervention against whose outcomes the study groups’ test scores could be juxtaposed. 

Secondly, the VR devices applied in this study (i.e., Carl Zeiss goggles) had not been referenced in most other studies as the training equipment of choice for the fully immersive VR environment. Only one study made use of the Carl Zeiss goggles [19], even though it focused on applying the Oculus Rift or HTC Vive models, so attempting any comparison of the respective results may not really be feasible in terms of applicable scientific rigour. The above-referenced models are not fitted out with the hand controllers, to the effect that any body movement has to be initiated by the appropriate movement of the subject’s head, as well as by having his eyes focused on a specific point in space. 

On the other hand, this ostensible study limitation at the same time happens to reveal the clear potential for making use of fully immersive VR training equipment of perceptibly lesser technological sophistication, which may well prove comparably effective in training older adults whilst boasting the added advantage of more affordable pricing. 

## 5. Conclusions

Training in a fully immersive VR environment, combined with a scope of dual-task activities (VR + DT), clearly improved individual balance. The VR-assisted physiotherapy should best complement the conventional physiotherapy regimens already in use (e.g., OTAGO programme). The benefits of VR solutions indicate that older women should make extensive use of this form of rehabilitation activity on a regular basis, preferably under the guidance of a therapist, unless specifically contraindicated.

## Figures and Tables

**Table 1:** Homogeneity analysis of the study groups.

Characteristics	Total (N = 80)	OTAGO Group (n = 40)	VR + DT Group (n = 40)	χ^2^ or U ^b^	*p*-Value
Age (years), mean (SD)	76.73 (1.99)	76.75 (1.77)	76.70 (2.21)	0.52	0.59
Gender (woman), n (%)	80 (100.00)	40 (50.00)	40 (50.00)	0.00	1.00
Education (years), mean (SD)	11.38 (3.47)	11.03 (3.34)	11.73 (3.61)	2.11	0.91
Body weight [kg] mean (SD)	73.19 (11.69)	75.68 (11.83)	70.70 (11.14)	1.84	0.07
Height [m] mean (SD)	1.67 (0.08)	1.68 (0.09)	1.66 (0.07)	1.26	0.20
BMI [kg/m^2^] mean (SD)	26.09 (2.41)	26.94 (2.51)	25.61 (2.23)	1.81	0.07
Number of persons who sustained a fall within the last year n (%)	19 (21.59)	8 (20.00)	11 (27.50)	0.62	0.43
Number of medications taken regularly mean (SD)	4.85 (2.30)	5.15 (2.21)	4.55 (2.36)	1.01	0.27
MMSE (pts) mean (SD)	26.74 (1.76)	26.47 (2.02)	27.18 (1.38)	−0.87	0.37
BBS (pts) mean (SD)	39.50 (1.16)	39.33 (0.94)	39.68 (1.33)	−1.49	0.11
TUG (s) mean (SD)	13.73 (1.41)	13.45 (1.06)	14.02 (1.66)	−1.33	0.18
TUG MAN (s) mean (SD)	13.47 (1.21)	13.72 (1.24)	13.23 (1.14)	1.77	0.08
TUG COG (s) mean (SD)	16.59 (2.75)	16.16 (1.67)	17.02 (3.48)	−0.96	0.34
SLS OP (s) mean (SD)	7.01 (6.63)	5.39 (1.56)	8.81 (8.99)	−0.98	0.33
SLS CL (s) mean (SD)	1.46 (1.23)	1.40 (0.85)	1.52 (1.54)	0.34	0.73

Abbreviations: SD—Standard Deviation; MMSE—Mini-Mental-State Examination, BBS—Berg Balance Scale; χ^2^—Pearson’s chi-square test ^b^; U Mann-Whitney test; TUG—timed up and go; TUG COG—timed up and go cognitive; TUG MAN—timed up and go manual; SLS OP—single-leg stance open eyes; SLS CL—single-leg stance closed eyes.

**Table 2:** Balance test results in both study groups (2nd measurement point).

Characteristics	Total (N = 80)	OTAGO Group (n = 40)	VR + DT Group (n = 40)	U ^b^	*p*-Value
TUG (s) mean (SD)	11.98 (1.36)	11.35 (1.09)	12.60 (1.34)	−3.96	<0.001
TUG MAN (s) mean (SD)	12.02 (1.38)	12.42 (1.57)	11.62 (1.02)	1.84	0.06
TUG COG (s) mean (SD)	14.37 (1.82)	14.60 (1.46)	14.13 (2.11)	0.41	0.68
SLS OP (s) mean (SD)	8.88 (6.99)	7.90 (4.94)	9.87 (8.52)	−1.47	0.14
SLS CL (s) mean (SD)	2.11 (1.03)	2.07 (0.71)	2.33 (1.27)	−0.94	0.34
BBS (pts) mean (SD)	42.23 (2.63)	42.58 (2.79)	41.88 (2.44)	0.01	0.99

Abbreviations: SD—Standard Deviation; χ^2^—Pearson’s chi-square test ^b^; U Mann-Whitney test; TUG—timed up and go; TUG COG—timed up and go cognitive; TUG MAN—timed up and go manual; SLS OP—single-leg stance open eyes; SLS CL—single-leg stance closed eyes; BBS—Berg Balance Scale.

**Table 3:** Balance test results in both study groups (3rd measurement point).

Characteristics	Total (N = 80)	OTAGO Group (n = 40)	VR + DT Group (n = 40)	U ^b^	*p*-Value
TUG (s) mean (SD)	12.54 (0.98)	12.26 (0.75)	12.82 (1.11)	−2.74	0.01
TUG MAN (s) mean (SD)	12.93 (2.08)	12.75 (1.32)	13.12 (2.64)	0.35	0.73
TUG COG (s) mean (SD)	15.01 (1.25)	14.88 (1.19)	15.15 (1.31)	−0.99	0.32
SLS OP (s) mean (SD)	7.96 (6.92)	6.92 (4.50)	9.00 (8.64)	−1.39	0.16
SLS CL (s) mean (SD)	2.00 (1.06)	1.91 (0.74)	2.08 (1.32)	−0.16	0.87
BBS (pts) mean (SD)	41.85 (2.70)	42.33 (2.94)	41.38 (2.36)	0.89	0.36

Abbreviations: SD—Standard Deviation; χ^2^—Pearson’s chi-square test ^b^; U Mann-Whitney test; TUG—timed up and go; TUG COG—timed up and go cognitive; TUG MAN—timed up and go manual; SLS OP—single-leg stance open eyes; SLS CL—single-leg stance closed eyes; BBS—Berg Balance Scale.

**Table 4:** Comparison of the results in respective measurement points, mean (SD).

		VR + DT Group				OTAGO Group		
	Pre- Intervention (n = 40)	Post- Intervention (n = 40)	After 3 Weeks (n = 40)	*p*-Value	Pre- Intervention (n = 40)	Post- Intervention (n = 40)	After 3 Weeks (n = 40)	*p*-Value
TUG (s) mean (SD)	14.02 (1.66)	12.60 (1.34)	12.82 (1.11)	0.001	13.45 (1.06)	11.35 (1.09)	12.26 (0.75)	<0.001
TUG MAN (s) mean (SD)	13.23 (1.14)	11.62 (1.02)	13.12 (2.64)	0.001	13.72 (1.24)	12.42 (1.57)	12.75 (1.32)	<0.001
TUG COG (s) mean (SD)	17.02 (3.48)	14.13 (2.11)	15.15 (1.31)	0.001	16.16 (1.67)	14.60 (1.46)	14.88 (1.19)	<0.001
SLS OP (s) mean (SD)	8.81 (8.99)	9.87 (8.52)	9.00 (8.64)	0.01	5.39 (1.56)	7.90 (4.94)	6.92 (4.50)	0.001
SLS CL (s) mean (SD)	1.52 (1.54)	2.33 (1.27)	2.08 (1.32)	0.001	1.40 (0.85)	2.07 (0.71)	1.91 (0.74)	<0.001
BBS (pts) mean (SD)	39.68 (1.33)	41.88 (2.44)	41.38 (2.36)	0.001	39.33 (0.94)	42.58 (2.79)	42.33 (2.94)	<0.001

Abbreviations: SD—Standard Deviation; TUG—timed up and go; TUG COG—timed up and go cognitive; TUG MAN—timed up and go manual; SLS OP—single-leg stance open eyes; SLS CL—single-leg stance closed eyes; BBS—Berg Balance Scale.

## Data Availability

The datasets generated and/or analysed during the current study are available from the Corresponding Author upon reasonable request.
